# Advances in genome studies in plants and animals

**DOI:** 10.1007/s10142-014-0364-5

**Published:** 2014-03-14

**Authors:** R. Appels, J. Nystrom-Persson, G. Keeble-Gagnere

**Affiliations:** Veterinary and Life Sciences, Murdoch University, 90 South Street, Murdoch, Perth, WA 6150 Australia

**Keywords:** Plant genomics, Animal genomics, Human genomics, Biological processes, Computer analyses

## Abstract

The area of plant and animal genomics covers the entire suite of issues in biology because it aims to determine the structure and function of genetic material. Although specific issues define research advances at an organism level, it is evident that many of the fundamental features of genome structure and the translation of encoded information to function share common ground. The Plant and Animal Genome (PAG) conference held in San Diego (California), in January each year provides an overview across all organisms at the genome level, and often it is evident that investments in the human area provide leadership, applications, and discoveries for researchers studying other organisms. This mini-review utilizes the plenary lectures as a basis for summarizing the trends in the genome-level studies of organisms, and the lectures include presentations by Ewan Birney (EBI, UK), Eric Green (NIH, USA), John Butler (NIST, USA), Elaine Mardis (Washington, USA), Caroline Dean (John Innes Centre, UK), Trudy Mackay (NC State University, USA), Sue Wessler (UC Riverside, USA), and Patrick Wincker (Genoscope, France). The work reviewed is based on published papers. Where unpublished information is cited, permission to include the information in this manuscript was obtained from the presenters.

## Introduction

**Fig. 1:**
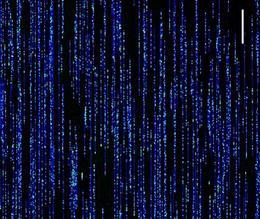
The BioNano technology builds on the earlier optical mapping technologies (Lin et al. 2012) by loading the DNA into nanochannels so that the DNA can be easily scanned to provide DNA fingerprints of molecules at least 500 kb or more in length (*bar at the top right* is 100 kb). The DNA map is compiled using two nicking enzymes, Nt.BbvCI and Nt.BspQI, followed by labeling the nick motifs (using DNA polymerase) with red and green dyes, respectively. The image, with labeling at the Nt.BspQ1 sites, is unpublished and was kindly provided by H. Van Steenhouse (BioNano, San Diego) based on DNA from the short of wheat chromosome 7D provided by H. Simkova and J. Dolezel (Czech Republic)

The analysis of genomes has been accelerated by advances in new technologies, such as the high-throughput sequencing of whole genomes, which provides an extensive view of the gene space of organisms. Large-scale sequence-based comparative studies have defined conserved and variable (conditionally dispensable, reviewed in Appels et al. 2013) regions of the genome as well as single nucleotide polymorphisms (SNPs) for molecular markers. The rapid development of DNA sequencing technologies over the last 5 years has meant that large genomes previously out of reach can be subjected to sequencing at a relatively low cost. However, cost-effective and high-throughput techniques for obtaining long-range information about the DNA sequence are still not widely deployed (Selvaraj et al. 2013). Mate–pair libraries can typically be taken up to 20 kb, with 40 and 150 kb inserts possible with fosmid and bacterial artificial chromosome (BAC) clones, respectively. Although physical mapping and assembly of BAC clones still form the backbone of most reference-level assemblies, ordering and orientation of the physical contigs within the genome as a whole remain a challenge (Selvaraj et al. 2013; Burton et al. 2013). The need for long-range mapping of sequences in complex genomes arises because of the need to deal with the ambiguities generated by the presence of extensive tracts of retro-transposable elements. In the genomes of plants such as wheat, the long-range mapping of BAC libraries using KeyGene (van Oeveren et al. 2011; Bayer Crop Science 2013) and BioNano (Lam et al. 2012; Hastie et al. 2013) technologies is providing the basis for more accurate sequence assemblies. The BioNano technology has also been applied to genomic DNA from flow-sorted chromosome arms of wheat 7D (short arm), and it is evident (Fig. 1) that DNA molecules up to and exceeding 500 kbp can be fingerprinted using this approach.

High-resolution genetic maps are now also providing genuine contributions to the assembly of complex genomes. In gene-rich regions, the whole genome sequencing of segregating progeny from a cross between two individuals (Mascher et al. 2013) provides thousands of SNPs for the generation of genetic maps with markers spaced less than 10 kb apart (provided the number of segregating individuals analyzed is sufficiently large, Cavanagh et al. 2013; Wang et al. 2014). Even where SNP markers co-segregate, the respective genome sequence contigs that are marked by the SNPs can then be reexamined with the additional knowledge that they are physically close together. In regions where genetic recombination is low (near the centromere), radiation mapping (Feuillet et al. 2012) has provided data contributing to the genome assembly pipeline. The assembly of complex genomes thus integrates diverse sources of data and requires information structure that moves well beyond the requirements of whole genome sequencing per se in the assembly of small genomes. As noted by Burton et al. (2013), these developments for complex genomes follow the model established by the Human Genome Project which integrated a diversity of approaches to achieve the long-range contiguity currently available in the reference genome. The establishment of a good reference genome in model systems, and for the human genome, has provided the foundation for many of the studies reported in this mini-review.

## Translating genome and post-genome information to society and industry

Genomic features that were discovered very early and used in diagnostic assays focused on repetitive sequence regions (Appels et al. 1978). The regions appeared to be species-specific in crop plants, and in human forensics, the regions provided the high levels of polymorphism required for distinguishing between members of a family (Jeffreys et al. 1985). The finding of a 33-bp sequence that was repeated in an intron of the myoglobin gene was flanked by a 9-bp direct repeat similar to target site duplications created by transposable elements (Jeffreys et al. 1985) led to the finding that these repeated regions are more widespread in the human genome. Although the direct sequence repeats flanking the short repetitive sequences (mini-satellites) proved not to be a general feature, the mini-satellites are widespread and share a core, conserved, sequence that can be readily assayed. The high levels of recombination at these repetitive sequence loci lead to unequal crossing-over and hence to variation in repeat numbers and levels of polymorphisms that far exceed variation due to mutation alone. In his plenary lecture, John Butler noted that the mini-satellite marker systems have stood the test of time and the high levels of polymorphisms continue to contribute to the DNA profile database that assays a set of 15 simple tandem repeats (STRs). Eight of these STRs overlap with those used in Europe. The database of 13 million DNA profiles (http://www.fbi.gov/anout-us/lab/biometric-analysis) is deployed in crime scene investigations, accident victim and soldier identification, paternal testing, immigration, and missing person investigations as well as providing a reference point for identifying convicted felons (Biesecker et al. 2005).

John Butler noted that although new genome sequence information can provide additional markers for diagnostics (Hares 2012), the value of the current suite of markers is in the legacy information and the ability to relate any new developments to the established suite of markers. In special situations, specific SNPs for distinguishing between identical twins, eye color (Yun et al. 2014), or a new suite of STRs to identify Y chromosomes have been established (PowerPlex Y23, Coble et al. 2013). The importance of multi-allelic markers was the key feature for markers in forensic science, and this has been expanded to plants (Nybom et al. 2014) and domesticated animals (http://www.nfstc.org/pdi/Subject09/pdi_s09_m01_04_a.htm).

In her plenary lecture, Elaine Mardis focused on the cancer genome and the design of diagnostics and vaccines for clinical use (Mardis 2010). The Cancer Genome Atlas (TCGA) network of groups collaborates to compile the molecular details of large numbers of human tumors (TCGA et al. 2013), aiming to have 10,000 specimens analyzed from 25 different tumor types by 2015. The TCGA Pan-Cancer analysis focuses on the details of tumor types at the subtumor/single-cell level in order to define the different consequences of changes in the expression of similar genes and build a gene network-based view of the cancer phenotype. The across-tumor comparative analyses are argued to provide a valuable path into understanding the genetic and epigenetic backgrounds of cancer.

Classical studies on cancers deployed whole chromosome analyses to characterize translocations. In some instances such as chronic myelogenous leukemia (CML), the gene fusion generated as a result of the breakage–rejoining event underpinning the translocation formed a new oncogenic BCR-ABL gene fusion (Melo 1996). Whole genome sequencing provides greater sensitivity for defining translocation events (Chen et al. 2013), and this was illustrated by Welch et al. (2012) in their analysis of a case of acute promyelocytic leukemia (AML) where no chromosomal abnormalities were cytologically visible. The genome-level analysis indicated that a 77-kb segment from chromosome 15 was translocated into the second intron of the *RARA* gene to create a new fusion gene that was expressed in the leukemia. Importantly, the study allowed new molecular probes to be designed to assay events of this type in other patients. Analysis of AML more widely has also shown that heterogeneity in tumors can provide the basis for relapse due to clonal selection of chemotherapy-resistant cell types resulting from new mutations (Ley et al. 2008; Ding et al. 2012). Remissions or poor response to radiation treatment in acute lymphoblastic leukemia (ALL) can also reflect modified double-strand break repair pathways (Marston et al. 2009). Transcriptome-based studies have provided molecular markers to identify possible pathways that should be inhibited in order to increase the efficacy of radiation treatment (Marston et al. 2009).

Glioblastoma (GBM) was the first cancer type to be systematically studied by TCGA (Brennan et al. 2013). The analysis of the cancer pathways in GBM has been facilitated by whole-exome and transcriptome sequencing and indicated that in 85.3 % of the tumors, the regulation of the p53 pathway was disrupted through mutation/deletion of TP53 (27.9 %), amplification of MDM1/2/4 (15.1 %), and/or deletion of CDKN2A (57.8 %). The depth of analysis also included proteome-level studies and indicated that the impact of genome change on the proteome is not always consistent with expectations and had significant implications for the clinical applications of the knowledge base. The analyses by Brennan et al. (2013) also highlighted the importance of having several time points available in an analysis in order to map the changes in the genome and proteome of different cell types comprising the tumor as it develops over time. A database which complements the outputs from TCGA is DGIdb (Griffith et al. 2013; http://dgidb.org/) and provides the capability of screening gene translations against drug–gene product interactions in order to translate research findings into clinical outputs. The database allows genes of interest to be grouped with genes for which drugs have already been developed. Another important output from proteome-level information is that it provides the capacity to identify unusual antigens that are unique to cells throughout the tumor and bind to MHC molecules. The sequences of these antigens can then be used to design peptides for raising vaccines that target specific tumors (Waldmann 2003; Restifo et al. 2012a, b).

## Dynamics of genome change and the distribution of variation

The plenary lecture by Sue Wessler examined the changes to the genome as a result of transposable elements (TEs) with a particular focus on a 430-bp miniature inverted repeat transposable elements (MITEs) called mPing. The mPing elements are a class 2 element (Wicker et al. 2007; Han et al. 2013) in the PIF/Harbinger superfamily, and in some rice cultivars, the element has reached high copy numbers (Naito et al. 2006, 2009). The Ping element contains the genes required for transposition (ORF1 and a Transposase, TPASE; Naito et al. 2009), and while these have been deleted in the mPing element, the genes are still required for mPing transposition. The mPing element was reported to undergo bursts of amplification and, based on the analysis of over 1,700 insertion sites in rice, was preferentially located within 1 kb upstream in the 5′ region of transcription start sites or within 1 kb downstream from the 3′ stop site (Naito et al. 2009). This preferential location close to genes was also found for other MITE families in *Brachypodium*, sorghum, and maize (Han et al. 2013). In transgenic experiments, the transposition of mPing in *Arabidopsis* utilized transposases from either autonomous Ping or Pong elements, or the cDNA from a Ping transcript (Yang et al. 2007). Most mPing excision sites are repaired accurately in *Arabidopsis* (Yang et al. 2007).

The preferential insertion of mPing elements into the promoters of rice rather than exons means that their effects on gene expression may be more subtle in modifying gene expression and changing network interactions. The basis for the preferential insertion of mPing elements was investigated further by introducing mPing elements (plus a Ping cDNA containing the ORF1 and TPase coding regions) into soybean (Hancock et al. 2011). The analysis of 72 insertion sites and the determination of their location within 5 kb of a gene annotated in the soybean genome sequence indicated that in rice, the preferential insertion observations were the result of an avoidance of exon insertion. The analysis of the soybean data indicated that mPing did not avoid exon insertions. The mPing element prefers to insert into a 9-bp sequence that is AT-rich, and thus, because rice exons are GC-rich, the avoidance of exon insertions is more obvious than in soybean where the exons are less GC-rich. This means that the avoidance of rice exons can be attributed to their high GC content and probably contributes to the ability of this element to attain high copy numbers despite a preference for inserting near genes. It has been speculated that this preference for insertion near genes may reflect interactions between the transposition machinery and some features of chromatin structure such as compactness/density of nucleosomes (Hancock et al. 2011).

The varieties of rice that contain the most actively transposing element (Naito et al. 2006) are evidently the result of independent and ongoing bursts of mPing amplification for over at least a century. In terms of the dynamics of genome structure, research is ongoing to study how TEs with a preference for inserting near genes can attain high copy number (hundreds, thousands of elements) without killing their host and how these TEs accumulate without being detected by host genome surveillance.

Relating variation in the genome and gene expression to observed phenotypes in populations of *Drosophila* was addressed by Trudy Mackay in her plenary lecture. A key resource, the *Drosophila melanogaster* reference panel (DGRP) provided a template for genotype–phenotype mapping (Mackay et al. 2012), and the presentation gave an update on DGRP activity with non-SNP variants being called and genome-prediction methodologies being improved. The panel comprised 192 inbred strains derived by utilizing mated females from an outbred population plus 20 generations of full-sibling inbreeding. The DGRP thus contains a representative sample of genetic variation and, based on the resequencing of 168 lines, has a high-resolution genetic map for locating phenotypic variation in resistance to starvation stress, chill coma recovery time, and startle response.

The resource provided 2,490,165 SNPs and 77,756 microsatellites (minor allele present in at least four lines) for associating chromosome regions to phenotypes of interest. As a proof of concept, the genes *Sema-1a* and *Eip75B* were found to be associated with startle response, and pnt with starvation resistance, consistent with previous studies. Against this background, the suite of new candidate genes identified has formed targets for new research (Mackay et al. 2012).

The outputs from the DGRP analysis have been compared to studies carried out on an advanced intercross population derived from 40 DGRP lines randomly mated as a large population size for over 70 generations (Flyland, Huang et al. 2012). The Flyland population was analyzed using extreme pools for starvation resistance, startle response, and chill coma recovery, and among the segregating SNPs in the DGRP, 1,339,448; 1,605,264; and 1,406,458 were segregating in the respective pools from the Flyland population. It was striking that none of the SNP associations in the Flyland population proved to be significant in DGRP (Huang et al. 2012), and further analysis showed that this was due to widespread epistatic interactions affecting all three traits (Mackay 2014), which could be identified because of differences in allele frequency between the DGRP and Flyland populations.

Mutations due to P elements (transposable elements in *Drosophila*; Magwire et al. 2010) and inversions (Corbett-Detig and Hartl 2012; Mackay et al. 2012) have been characterized in the DGRP as well as in other populations. As many as 40 % of the 1,332 P elements assessed affected traits, one third affecting life span, and among these, 58 were associated with increases in life spans (Magwire et al. 2010). The inversions corresponded to regions of increased diversity (Corbett-Detig and Hartl 2012; Mackay et al. 2012) although in some instances, inversions of 1.7 Mb showed no polymorphisms, probably because they have arisen relatively recently (Corbett-Detig and Hartl 2012). Inversions tended not to disrupt gene sequences. Where the ends of the inversion do interrupt gene/transcribed sequences, it is correlated with the finding that these inversions have short inverted duplications at their ends. It is evident that inversions and transposable elements are a significant source of variation in chromosome structure within a species. The inversion-level structural variation in the genome has a more immediate significance within an organism such as *Anopheles gambiae*, where the variation due to inversions is considered to be a basis for the success of the mosquito adapting to regions in North Africa (Lee et al. 2013).

The quantitative regulation of gene expression with a focus on individual cells in a tissue was studied at a specific locus in *Arabidopsis* by Caroline Dean in her plenary lecture. The locus for the primary control of flowering in *Arabidopsis* (and other flowering plants) is Flowering Locus C (FLC) and codes for a MADS-box domain protein that represses flowering (Song et al. 2013). Inactivation of the repressor by a prolonged period of low temperature (vernalization) allows flowering to occur when the environmental temperature increases. For example, if only 2 weeks of cold was experienced, flowering is later than if 4 weeks of cold occurred (Song et al. 2013). This quantitative response was shown to result from individual cells in the flowering meristematic tissue switching between epistatic states and that it was the proportion of cells that were switched that gave the overall quantitative response.

The mechanism for establishing the level of FLC expression characteristic for a variety adapted to a given environment is based on the conserved chromatin modifiers (reviewed in Crevillén and Dean 2011). The recruitment of the chromatin modifiers to the promoter scaffold of the FLC locus is enhanced by the FRIGIDA protein complex (FRI; Choi et al. 2009) by directly interacting with the nuclear cap-binding complex required for RNA transport/stability. Variation in FRI has been associated with adaption to different environments, and this correlates with FRI influencing the proportion of FLC RNA with a 5′ cap (Geraldo et al. 2009). The chromatin structure at the FLC locus is such that the 5′- and 3′-flanking regions of the gene form a chromatin loop (Crevillén et al. 2013) as part of the active locus conformation. In order to silence the FLC locus, the influence of the chromatin modifiers is reversed by reducing H3 histone methylation at lysine 4 and 36 and increasing methylation at lysine 27. These changes in methylation are carried out in combination with the alternative splicing and polyadenylation of antisense transcripts (COOLAIR) downstream from the polA site and within the chromatin loop (Swiezewski et al. 2007, 2009; Angel et al. 2011; Liu et al. 2007; Crevillén et al. 2013). A key variable in the level of expression of FLC is the level of methylation at H3 histone lysine 27.

At the level of individual cells in the flowering meristem tissue, it is evident that the switch between FLC on or off is not a quantitative effect. Instead, it is the proportion of cells that are switched on which defines the overall response of the meristem to the start of flowering (Rosa et al. 2013). The importance of RNA-based regulation of gene expression, at a more general level, was emphasized by Caroline Dean since 50 % of annotated transcripts have low levels of antisense transcripts which could function in a way that is analogous to the COOLAIR-based modification of chromatin structure at the FLC locus. The sensing mechanisms for cold are still under investigation and are likely to comprise a number of different networks within the plant (Miura and Furumoto 2013).

## Integrating large datasets

The requirement for information structure in biology was discussed by Ewan Birney in his plenary lecture and emphasized the importance of integrating diverse datasets as well as the interface between life sciences and industry/environment. His argument was based on cost-effectiveness particularly with respect to the development of diagnostics in health care and agriculture as well as marker technology associated with complex traits in the characterization and breeding of domesticated animals and plants. Information structure can lead to cost-effectiveness both through reduced hardware requirements and through a reduced need for manual labor in data analysis.

The long-term storage of biological datasets has raised questions about efficient algorithms, formats, and methods for data entry, curation, and retrieval (Church et al. 2012; Goldman et al. 2013). Generally, there exists a need to retain large datasets over extended periods of time and data compression formats have been investigated (Fritz et al. 2011). Compression formats can be grouped into *lossless* formats, from which a perfect copy may be restored, and *lossy* formats, which achieve better compression at the expense of the ability to restore the original in full detail. The bulk of high-throughput sequencing data, for example, is made up of sequencing reads and the corresponding quality scores, and in the case of *lossy* compression, it is often the latter that are sacrificed to some degree. In many cases, this has been shown to not compromise the usefulness of the data for subsequent downstream analysis (Popitsch and von Haeseler 2013). Data compression has been studied extensively in the field of computer science; however, in bioinformatics, the challenges are novel because extra assumptions can be made about the data, particularly in using the redundant nature of DNA information. DNA sequences have only four bases, and large sequencing datasets are often highly redundant and compression can be achieved by encoding only their difference when compared to a reference (Fritz et al. 2011).

Widespread access to larger storage capacity (for example ELIXIR, http://www.elixir-europe.org/, with a capacity of 35 PB and transfer speed of 40 GB/s) for a variety of data that needs to be publically available, shared between research groups, and integrated across different projects has made the links between such databases increasingly important. Metadata or “data describing the data” provides the information necessary for relating datasets to each other, to enable the respective dataset to be positioned within a particular research project (Rocca-Serra et al. 2010). The quality of the metadata can determine the possible ways a research project can utilize the data without direct access to the dataset’s original producers. It is now common for large, curated data repositories to standardize the way information is accessed with web services and query languages. The emerging standard for biological experimental metadata is the investigation, study, and assay (ISA) framework (http://www.isacommons.org). The generality and flexibility of tools such as the ISA framework makes it feasible to deal with the dual nature of data in terms of distinguishing between data and metadata. For example, much of the UniProt protein database is an accumulation of experimental data per se, and from this perspective, it is data. However, experimental data being released from other projects can be annotated with the relevant UniProt identifiers in order to provide a context for understanding and integrating new data, and from this perspective, the UniProt information becomes metadata. Thus, the data/metadata distinction is often relative to the main focus of a given experiment.

At the simplest level, two databases can be related to each other when they both reference a common set of biological variables. Some standards have emerged as de facto backbones for reference-based integration. For example, REACTOME cross-references UniProt, ChEBI, and Gene Ontology (GO); the PRIDE database cross-references UniProt and many other protein databases; and the Expression Atlas cross-references Ensembl genes, UniProt, and GO. Therefore, at the most basic level, a biology analyst can manually integrate data from several databases. However, for more complex integration analyses, special-purpose query languages are required to make it more straightforward to carry out the integration process. One approach that has been gaining momentum is the use of linked data/semantic web technologies such as Resource Description Format (RDF), which is often used together with the query language SPARQL. A key benefit of these formats is their open-ended nature and the fact that they are fundamentally designed for meaningful integration of heterogeneous data. Well-established databases providing their data as RDF include GO (http://www.geneontology.org), UniProt (http://beta.spargl.uniprot.org), and Bio2RDF (http://www.bio2rdf.org). In late 2013, EMBL-EBI embraced the RDF format in the EMBL RDF platform (http://www.ebi.ac.uk/rdf/). A closely related trend is the increasing support for programmatic (API) access to integrated databases. One example is the Proteomics Standard Initiative Common QUery InterfaCe (PSICQUIC), developed by the Human Proteomics Organization Proteomics Standards Initiative (HUPO-PSI) as a standard interface to molecular interaction data. This standard is specific to molecular interaction data, unlike RDF, which is fully general.

One of the challenges of effective metadata use is ensuring that related fields are comparable between experiments. For example, precisely defining the tissue type of a biological sample is nontrivial and definitions may differ between countries. The use of controlled ontologies solves this problem and provides a way for domain experts to describe their knowledge in a way that can be applied automatically for the purpose of integrating datasets. The GO is perhaps the most well known; other examples include the Plant Ontology (PO) and the Environmental Ontology (EnvO). The OWL framework, which is an extension built on top of RDF, has become one of the de facto standards for ontology development and use. The Open Biomedical Ontology (OBO) project has played a leading role in advancing the development of ontologies (http://www.obofoundry.org).

Procedures for data release are now starting to reflect the importance of data structure as exemplified by the open journal *GigaScience* (http://www.gigasciencejournal.com/), closely affiliated with BGI-Shenzhen (Ling 2013). *GigaScience* has a special category of paper, the “Data note,” which allows for the release of significant datasets. This ties in with the overall goals of DataCite (http://www.datacite.org), an international consortium of organizations aiming to support the use (and re-use) of public scientific data and to promote data as a research output in and of itself (Brase et al. 2009). It has been argued that publications for which data is made publicly available have a higher citation rate on average (Piwowar and Vision 2013). Well-established journals such as *Nature* are beginning to adopt data-publishing platforms as a core element of the publishing process. Importantly, digital object identifiers (DOIs), which have a long history of use in the context of scientific articles, are now being used to identify published datasets. It is now considered good practice for journal articles to cite the dataset used in the study in the references.

## Metagenomics in the discovery of new life-forms

The integration of high-throughput sequencing into semi- or fully-automated data acquisition methods, new bioinformatics tools, and standardized data organization forms the basis for metagenomic projects that investigate the life-forms populating different parts of our environment. The data outputs can be stored in a structured format so that access is relatively simple (https://www.ebi.ac.uk/metagenomics/). The plenary lecture by Patrick Wincker described the Tara Oceans project (Karsenti et al. 2011; Hingamp et al. 2013) in which approximately 27,800 biological samples were collected from 153 ocean stations at three depths that were defined by approximately 13,000 measurements for detailed analysis. The DNA analysis of the metagenomes was carried out on organisms 0.2 to 1.6 um in size, from 17 samples collected at 13 sites. The focus for the study was on the abundance of nucleocytoplasmic large DNA viruses (NCLDVs) in the marine environment, and to achieve this, 16 NCLDV marker genes and 35 cellular marker genes (including the 18S rDNA variable region) were used to align the Roche-454 reads produced from DNA extracted from the samples. The metagenomic reads were utilized without prior assembly software analysis by the COMPAREADS software (Maillet et al. 2012).

The NCLDVs constitute a group of eukaryotic viruses that have an ecological role in the sea in contributing to the turnover of their unicellular hosts as well as causing diseases in animals. The data presented by Patrick Wincker showed that metagenomic sequence analyses can guide the discovery process for new marine viruses and their host interaction in future research.

## Directions for genomic research

The use of DNA as a storage medium for digital information as a replacement for hard drives is a future possibility being actively investigated, as discussed by Ewan Birney in his plenary lecture. Recently, a scalable method that encodes nontrivial amounts of information was described (Goldman et al. 2013). DNA is an attractive medium because of its proven durability and very high storage density that is several orders of magnitude greater than any existing commercial storage devices (Church et al. 2012). However, applications will not be economical until the cost of sequencing and synthesizing arbitrary DNA sequences has been greatly reduced.

The plenary lecture by Eric Green provided an overview of the broad area of genomics with a particular focus on the human genome (Green and Guyer 2011; McCarthy et al. 2013). It was evident that advancing genomics to attain complete reference genomes in order to define functional elements in the proteome was a top priority. The large scale of genomics required attention to organizational structure because international consortia are usually involved, data standards to minimize errors and maximize utility, and computational procedures. The rapid release of the large data catalogs was a priority but needed to respect the ownership by researchers and initiatives such as DataCite (Brase et al. 2009), discussed in this review and which introduced digital object identifiers (DOIs), were beginning to deal with the need for structure in the information becoming available. The requirement for low-cost data production (DNA, RNA sequencing, proteomics, and metabolomics) was closely linked to the translation of research outputs to society more broadly and the development of appropriate policies. Many of the concepts and challenges in human genomics also apply to plants and animals as discussed in this review.

In the human genomic area, application in cancer pharmacogenomics, the diagnosis of rare disorders, and the tracking of disease outbreaks are new applications that build on the developing expertise (McCarthy et al. 2013). Some breakthroughs in defining the molecular biology of well-known disorders have been provided in this review. In addition, the role of the host microbiome (through metagenomic capabilities) and identification of drug response biomarkers in order to facilitate the application of existing drugs for new purposes are targets for a broader translation into society. The application of DNA sequencing to providing a noninvasive test for prenatal screening (Allison 2013) is an example of a particularly rapid uptake. The approach is based on the discovery that cell-free fetal DNA (cffDNA) is released when placental cells break down, and this cffDNA can comprise 5–10 % of the genetic material in a pregnant woman’s bloodstream. The placental source of the DNA means false positives for trisomy of chromosomes are possible (since this tissue is not necessarily identical to that of the fetus), and the data is generally treated as an indicator of risk only (Allison 2013).

A corollary of the speed at which data acquisition is occurring is the need for education programs to keep up and provide the required training for the next generation of researchers, policy makers, and participants in industry and society more broadly. The new technologies need to be matched by a biological understanding within the broad range of researchers and clinicians (McCarthy et al. 2013) as well as individuals involved in extension and consulting activity in agriculture.

For the use of databases, Ewan Birney noted that EMBL/EBI had upgraded its infrastructure for online training (https://www.ebi.ac.uk/training/). In the medical area, both Eric Green and Elaine Mardis emphasized the requirements for clinicians to understand genomic-based information, as well as the importance of having access to the research outputs (http://www.iccg.org/; https://www.ncbi.nlm.nih.gov/clinvar/). In the plant and animal areas, innovation in undergraduate-level teaching (Burnette and Wessler 2013) is deploying the research outputs in the transposable element area as a model for students in biology to experience first-hand the analysis of phenotypes and genotypes. This provides the basis for correlating changes in genotypes using marker polymorphisms, for understanding the fundamentals of biology, as well as for appreciating the nature of research and applying knowledge from one organism to another.
